# Oxidative stress as a hidden cost of attractiveness in postmenopausal women

**DOI:** 10.1038/s41598-020-76627-9

**Published:** 2020-12-15

**Authors:** Urszula M. Marcinkowska, Anna Ziomkiewicz, Karel Kleisner, Andrzej Galbarczyk, Magdalena Klimek, Amelia Sancilio, Grazyna Jasienska, Richard G. Bribiescas

**Affiliations:** 1 Department of Environmental Health, Faculty of Health Sciences, Jagiellonian University Medical College, Kraków, Poland; 2 Department of Anthropology, Faculty of Biology, Jagiellonian University, Kraków, Poland; 3 Department of Philosophy and History of Science, Faculty of Science, Charles University, Prague, Czech Republic; 4 Department of Anthropology, Yale University, New Haven, Connecticut, USA; 5 Department of Anthropology, Northwestern University, Evanston, Illinois USA

**Keywords:** Human behaviour, Biological anthropology, Sexual selection

## Abstract

Perceived facial attractiveness, a putative marker of high biological fitness, is costly to maintain throughout a lifetime and may cause higher oxidative stress (OS). We investigated the association between the facial features of 97 postmenopausal women and their levels of OS biomarkers 8-hydroxy-2′-deoxyguanosine (8-OHdG), superoxide dismutase (Cu-Zn SOD), and thiobarbituric acid reactive substances (TBARS). In study 1, 966 judges rated the composites (facial averages) of women with higher OS as more attractive, healthier, younger, and less symmetric. In study 2, Geometric Morphometric analysis did not reveal significant differences in facial morphology depending on OS levels. In study 3, measured facial averageness and symmetry were weakly negatively related to 8-OHdG levels. Maintaining higher perceived facial attractiveness may be costly due to increased oxidative damage in the postmenopausal period. These costs may remain hidden during the reproductive period of life due to the protective mechanisms of oxidative shielding and revealed only after menopause when shielding has ceased.

## Introduction

### Why faces matter?

Facial recognition is crucial for daily human interactions. The biological basis of facial attractiveness has been studied intensely for a number of decades^1–3^ and it has been suggested that judgements of facial attractiveness are more important than those of the body when comes to sexual preferences^4^. Moreover, the importance of facial recognition is also visible in the very early onset of attention towards attractive faces in ontogenetic development^5, 6^.

Facial features were proposed to serve as a cue to health^7^, age^8^, mating success^9^, and sexual behavior^10^. However, it is still unclear why some faces are rated more attractive than others. Proposed facial features such as symmetry^11^, averageness^12^, and sexual dimorphism^13^ have all received mixed support. Nevertheless, it is well established that faces are of central importance for social interactions^5^ and that they convey numerous signals for the observer.

### Why does oxidative stress matter?

Oxidative stress (OS) is a consequence of normal aerobic cellular metabolism. It emerges from the imbalance between the production of reactive oxygen species (ROS), their utilization, and elimination via a range of enzymatically and non-enzymatically regulated antioxidative defense mechanisms. At low levels and in specific cells, ROS support several biological processes such as immune defense or cellular signaling. At high levels, ROS can cause damage to cellular lipids, proteins, and nucleic DNA. To avoid OS and alleviate the effect of high ROS concentrations, organisms have the ability to repair damage via non-enzymatic and enzymatic agents. Superoxide dismutase (SOD) is among the most potent enzymes that induce repair at the intercellular level.

Furthermore, non-enzymatic OS reducing factors such as carotenoids can be deployed from dietary sources to further support the repair process^14^. Interestingly, carotenoid-based ornamentation is a crucial factor that contributes to attractiveness in males and females of many animal species^15^. Recent studies also point to the significant effect of carotene-based pigmentation on the face and body attractiveness judgment in humans^16, 17^.

According to the Free Radical Theory of Aging, the reaction of active free radicals produced during metabolism within cellular constituents contributes to the aging process by causing cellular damage, which accumulates over time and impairs normal function^18^. Indeed, in humans, the level of oxidative damage increases with the advanced age and is partly responsible for decreased survival^19^⁠.

Individuals differ in their levels of oxidative stress over a lifetime depending on the behavior, physiological state, or environmental conditions. For women, investment in reproduction is associated with an increase in ROS production due to a significant rise in energy requirements. For example, increased oxidative status (higher levels of oxidation biomarkers) is observed in pregnant compared to non-pregnant and in pregnant multiparous compared to primiparous women^20^. These observations suggest that not only does oxidative stress increase during reproductive years, but it may also accumulate across consecutive bouts of reproduction^21^. Interestingly, levels of oxidative stress and the direction of the association between reproductive effort and oxidative stress during different stages of female life may differ between populations, perhaps due to differences in environmental stressors^22, 23^⁠.

### Association between oxidative stress and facial features

Perceived facial attractiveness has been suggested as an indicator of good health and overall biological fitness; however, evidence is mixed^24, 25^. Evolutionary approaches posit that preferences for certain traits evolved because those traits could provide a signal of biological quality. One such trait of biological quality is oxidative stress^26^. According to the “Handicap Principle”, any signal that honestly advertises high biological fitness is likely to create a cost to the signaler that would incur an undue burden for an individual with lower quality^27^. Schantz et al.^26^ proposed that OS might reflect a cost that arises from a trade-off between individual health, fitness, and the maintenance of condition-dependent ornamental sexual traits. Thus, maintaining these costly traits in stressful, energy-restricted environments should be associated with increased oxidative stress and perhaps compromised survival. Facial attractiveness might be considered such an ornamental trait or rather a group of traits that together indicate biological quality and might be linked to mating and reproductive success.

Results from the limited number of investigations on the association between oxidative stress and facial features within a context of signaling good health have been suggestive but often inconclusive. A study by Gangestad et al.^28^ found a modest negative correlation between male physical attractiveness and levels of two biomarkers of oxidative stress—8-OHdG (8-hydroxy-20-deoxyguanosine, a biomarker of damage to DNA), and MDA (malondialdehyde, a biomarker of lipid peroxidation). Levels of these biomarkers also predicted perceived healthiness, facial asymmetry, and masculinity. In contrast, Foo et al.^29^⁠ reported no associations between perceived facial attractiveness as indicated by facial sexual dimorphism, symmetry, averageness, and adiposity, and levels of 8-OHdG in women or men of reproductive age. Thus, although the relationship of oxidative stress to health and reproductive histories^30^, as well as between health and attractiveness^31^ are well established, it remains unclear how attractiveness is associated with oxidative stress.

### Current study rationale

This study aimed to investigate the association between women’s facial features (both perceived and measured) and oxidative stress as a reflection of somatic condition. None of the previous studies found significant associations between OS and women's perceived facial attractiveness. However, most of the participants in these studies were young and nulliparous^29^. As higher levels of OS markers typically occur as a result of aging and reproduction^22^, it is informative to base such analyses on a postmenopausal group of women as we did in this study.

The rationale behind the design and order of our studies was to examine the perception of faces with varying OS levels through the deployment of a large-scale online survey. We also conducted a follow-up analysis using a morphometric method and deployed additional face shape analyses to assess the role of facial symmetry and averageness. This approach allowed for the multidisciplinary and multidimensional assessment of the association between oxidative stress and face perception. By applying this approach, we hoped to gain greater insight into its complexity.

## Methods

### Study group

The data were collected from 97 postmenopausal women aged 48–92 (Mean = 65.5, SD = 8.89) living in a rural area of the Mogielica Human Ecology Study Site in southern Poland. This agricultural population is currently undergoing a demographic transition, however, for many decades it was characterized by natural fertility and high parity^32, 33^. High physical activity was common in this population as machinery usage was not possible due to the mountainous character of the terrain^34^. Also, self-food provisioning from the farmlands and livestock contributed to the high nutritional status of its’ inhabitants^35, 36^.

Trained study assistants visited the participants at home to collect personal data and perform anthropometric measurements (body height and weight) for body mass index (BMI) calculation. After an initial meeting, women were invited to the local health clinic, where they were examined by a medical doctor to screen potential participants for any current infections. Participants provided a first morning urine sample for analysis of oxidative stress biomarkers and also posed for facial photographs. All photographs were taken by the same researcher. None of the women exhibited facial injuries, facial malformations, facial modifications in the form of surgeries or piercings, fixed orthodontic appliances nor wore contact lenses. All methods were carried out in accordance with relevant guidelines and regulations. Written informed consent was obtained from every participant. The Jagiellonian University Bioethics Committee approved the protocol of the study.

### Oxidative stress biomarkers

Three biomarkers of oxidative stress were measured from first morning urine samples^22^. All analyses were conducted using commercial ELISA kits in accordance with manufacturer procedure in Yale Reproductive Ecology Lab by the same researcher (8-OHdG, Enzo Life Sciences, catalogue ADI-EKS-350; Cu-Zn SOD, Enzo Life Sciences, catalog ALX-850-033; TBARS, Cayman Chemical Company, catalogue 10009055).

8-OHdG is a modified nucleotide base and a by-product of repaired DNA damage that is excreted in urine. Levels of 8-OHdG depict the amount of oxidative damage accumulated in cellular DNA through repaired lesions on the guanine base pair caused by ROS. Cu–Zn SOD catalyzes the dismutation of superoxide anion radicals to free oxygen and hydrogen peroxide. Levels of Cu–Zn SOD represent a primary line of enzymatic defense produced in the cytoplasm to neutralize ROS. Levels of TBARS reflect peroxidation of cellular lipids resulting from oxidative stress.

The average inter-assay variability for 8-OHdG, Cu–Zn SOD, and TBARS analysis was 14%, and intra-assay variability was 5%. All results were corrected for urine concentration based on samples’ specific gravity measured with refractometer (model PAL-10S ATAGO, U.S.A., Inc).

### Visual stimuli creation

Composites of facial images were created using previously taken photographs of women^37^ using the PsychoMorph Program^38^. All original photos were taken using a Canon G12 camera, under standardized conditions of background and distance from the photographer. Twenty pictures were chosen (ages between 49.9 and 81.1 years old, Mean = 65.7 years, SD = 8.62) from all photographed and measured female participants to create two composite facial images: 10 for High Oxidative Stress Composite (HOS) faces and 10 for low oxidative stress composite (LOS). Original photographs forming two average composites were chosen based on ten photographs of women with the highest and ten photographs of women with lowest aggregated z-scored oxidative stress markers: 8-OHdG, Cu-Zn SOD, and TBARS (raw data available in Supplementary Material 1). These average composites are a visual representation of the mean distance between 230 landmarks on each base picture. Oxidative stress biomarkers had a normal distribution in only one of two composite groups (p < 0.01 in Kolmogorov–Smirnov test for 10 base photographs used to create HOS composite). Thus, the U Mann–Whitney test was used to test for the age difference between HOS and LOS groups. The average age of women in the base photographs did not differ between 2 composite pictures (Mean age HOS = 66.23 years, Mean age LOS = 65.01 years, Z = 0.34, p = 0.73). In contrast, all three markers of the OS differed (for all three OS markers U = 0.00, Z = -3.74, p < 0.001, see Tables S1, S2 in Supplementary Material 2).

From the 77 original facial images that remained after drawing 10 images for HOS and 10 images for LOS composites, a set of 10 new composites was created (each an average of 5 randomly chosen images). The composites were then transformed so that they resembled HOS or LOS averages. This was done by adding or subtracting 50% of the vector distances between all psychomorph landmarks. Individual facial images could not be presented to raters due to personal data protection. All transformed images were masked: the only face was visible, the background was black, no hair or ears visible.

### Study 1: perceived attractiveness

#### Procedure

Random judges (N = 966) completed an online questionnaire that consisted of socio-demographic section and a slide show depicting images of the transformed faces. The judges were predominantly women (90.1%) of European origin, with secondary or higher education and an average age of 29.9 years (SD = 7.75). Most of the judges (98%) declared being exclusively or predominantly heterosexual.

Participants were asked to choose a face they perceived as (i) healthier, (ii) more attractive, (iii) younger, and (iv) more symmetric using four randomized blocks, via 2-alternative forced-choice questions. Each block consisted of 10 questions depicting 2 transforms simultaneously: one resembling HOS composite, and one resembling LOS composite.

The effect of judges’ age on the odds of choosing a particular OS composite as more healthy, attractive, symmetrical, and younger was tested using correlation analysis. The difference between female and male judges in terms of their assessment was tested using the U Mann–Whitney test due to the unequal size of subsamples of female and male judges. Differences between the assessments of LOS and HOS composites with regard to health, attractiveness, youthfulness, and symmetry were tested in separate models of repeated measure analysis of covariance with judges' sex introduced as between-subject factor and judges' age as a covariant. The analyses were performed using the JASP version 0.10.2 (JASP Team 2019).

#### Results

A significant and positive association was found between judges’ age and the odds of choosing the LOS composite as healthier (r = 0.19, p < 0.001) more attractive (r = 0.17, p < 0.001), younger (r = 0.21, p < 0.001) but not more symmetrical (r = 0.002, p = 0.953). Furthermore, a significant effect of judges' sex was also found (Table S3 in Supplementary Material 2). In particular, male judges evaluated the LOS composite as healthier, more symmetrical, and younger, slightly more frequently than female judges.

**Figure 1:**
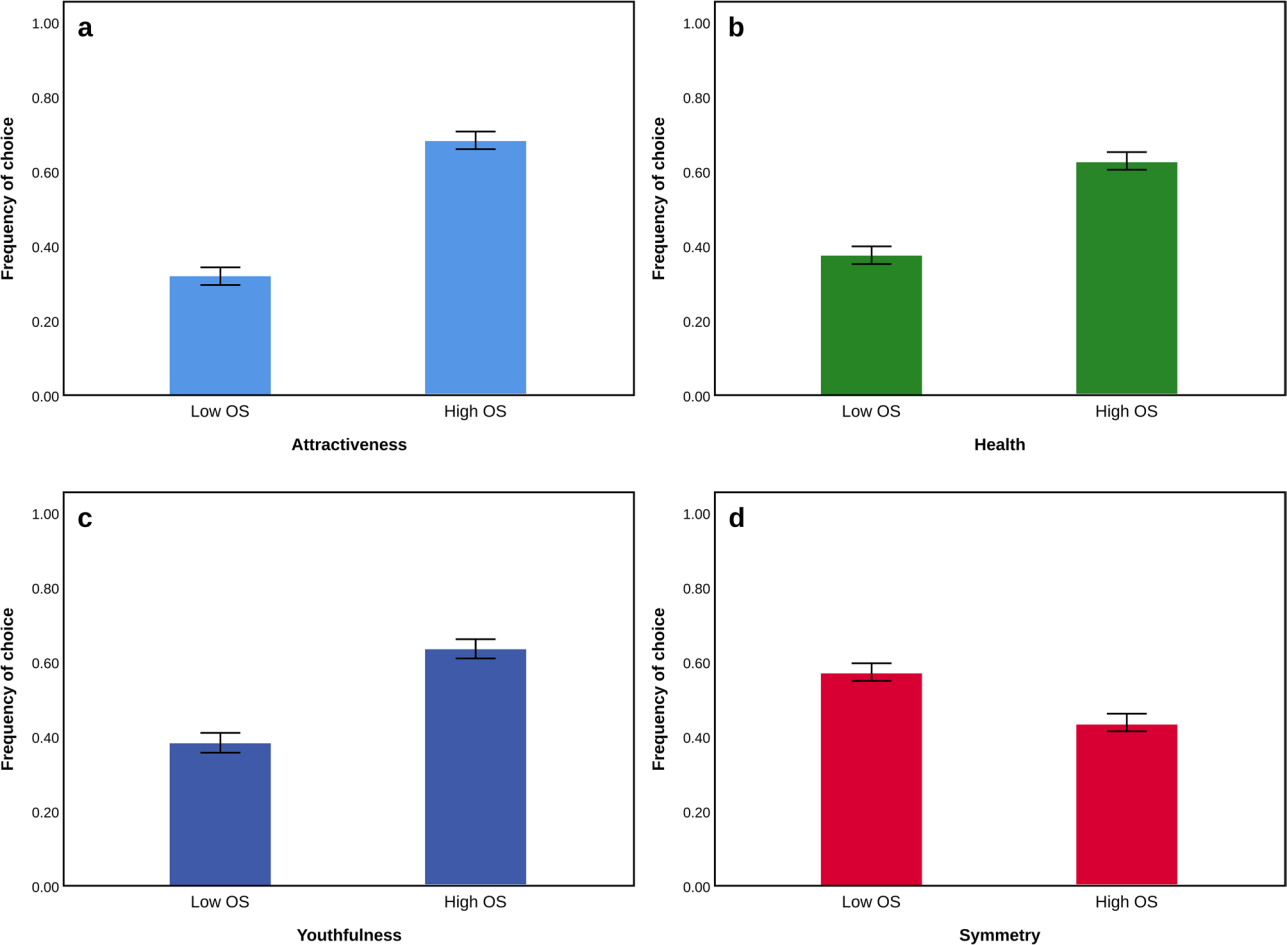
The frequency of choice of faces with low and high average levels of oxidative stress with respect to (**a**) attractiveness, (**b**) health, (**c**) youthfulness, and (**d**) symmetry. Differences between ratings are statistically significant at p < 0.001 (**a**–**c**), and at p < 0.05 (**d**).

HOS composites were chosen significantly more frequently than LOS composites as healthier (F = 84.39, p < 0.001), attractive (F = 111.98, p < 0.001) and younger (F = 88.78, p < 0.001) (Fig. 1). In contrast, LOS composites were chosen more frequently as symmetrical (F = − 4.370, p = 0.037) compared to HOS composites. The effect of judges’ age remained to be a significant covariant only in the model for attractiveness (F = 5.489, p = 0.019) but not for health (F < 0.001, p = 1.000), youthfulness (F = 1.582, p = 0.209) and symmetry (F = 3.510, p = 0.061). The effect of judges’ sex was not significant for all models (F < 0.001, p = 1.000 for health; F = 0.361, p = 0.548 for attractiveness; F = 0.068, p = 0.794 for youthfulness; F = 0.133, p = 0.715 for symmetry).

### Study 2: geometric morphometrics

#### Procedure

Using TpsDig2 software (ver. 2.31^39^), we placed 72 landmarks on each of the 97 facial photographs (taken from a previous study^37^), out of which 36 landmarks were denoted as semi-landmarks that describe curves and outline lining between true landmarks. For definitions and locations of landmarks and semi-landmarks, see our previous works^40, 41^. All shape coordinates (landmarks and semi-landmarks) were superimposed by Generalized Procrustes analysis using the ‘gpagen’ function in the Geomorph package in R^42^. Semi-landmark positions were optimized using a method that minimizes the bending energy between corresponding points. After the semi-landmarks were slid along the curves, the facial configurations were symmetrized along the bilateral axis^43^. Symmetrization was done in three steps (1) the x-coordinates of all landmarks were multiplied by − 1; (2) the bilaterally paired landmarks were re-labeled so that paired landmarks on the left side were given the labels of landmarks on the right side and vice versa, (3) the original and mirrored landmark configurations were averaged.

The possible effects of oxidative stress on facial morphology were investigated by multivariate regression analysis with the shape coordinates as a response variable and a particular measurement of oxidative stress (i.e., 8-OHdG, Cu-Zn SOD, TBARS) as a predictor. The p-values were estimated by permutation test based on 9,999 iterations. All multivariate regressions were performed using function "procD.lm" within R package geomorph^42^.

#### Results

The regression of symmetrized shape coordinates on either predictor revealed no direct influence of OS measures on facial morphology: 8-OHdG (F_(1,96)_ = 0.499 p = 0.882), Cu–Zn SOD (F_(1,96)_ = 0.513, p = 0.875), TBARS (F_(1,96)_ = 0.937, p = 0.449). When subject’s age and BMI were added to the model as covariates, the effect of OS on facial shape remained statistically non-significant.

### Study 3: bilateral symmetry and averageness

#### Procedure

We used non-symmetrized Procrustes residuals for all 97 female faces of our dataset. The facial coordinates (obtained via the procedure described in Study 2) were reflected along the vertical midline axis, and corresponding paired landmarks were re-labeled so that landmarks on the left side acquired the indexes of the landmarks on the right side and vice versa. As a measure of symmetry, we calculated a magnitude of vector difference between original and mirrored (reflected re-labeled) facial coordinates^44^. This corresponds to the Euclidean distance between mirrored and original configuration in face-space. The greater the distance between original and mirrored configuration, the less symmetrical (more asymmetrical) is the face.

The averageness was calculated as the Euclidean distance in shape space of each facial configuration to average landmark configuration. The greater the distance of a face from population average, the lower the averageness, i.e., lower values indicate a higher level of averageness. It is also highly probable that more asymmetric faces could be less average. Therefore, we calculated two measures of averageness using the original non-symmetrized shape coordinates (AVRG) and symmetrized shape coordinates (SAVRG). The first measure of averageness reflects all shape differences of a facial configuration from average, including those due to deviances in facial asymmetry. The latter measure (SAVRG) reflects only deviations from average in situations where all faces are ideally symmetric along the bilateral axis. The first approach (AVRG) better reflects the real-world situation where symmetry may contribute to averageness. The comparison of SAVRG and AVRG allows investigating whether the averageness that involves variation in bilateral symmetry has a stronger association with OS than the measure where symmetry contribution to averageness is filtered out.

The statistical association between facial symmetry (or averageness) and measures of OS were estimated by linear regression models using ‘lm’ function in R. The symmetry (or averageness) represented the dependent variable while the measures of oxidative stress were predictors. Participants’ age was also included in the initial models. The variables (facial symmetry and averageness) were log-transformed before linear modeling to eliminate the skewness of the data. Adjusted determination coefficients (R^2^) are reported as effect sizes.

#### Results

The results of the regressions of symmetry on measures of OS are presented in Table 1. Table 2 summarizes the results of regressions of both measures of averageness (AVRG, SVRG) on OS. When the relationship between symmetry or averageness and OS was controlled for age, age did not have a statistically significant effect (on either symmetry or both measures of averageness). Therefore, age was removed from the models.

**Table 1:** A relationship between symmetry scores and measures of oxidative stress tested by regression analyses.

	b (CI)	t	p	R^2^
8-OHdG	− 0.096 (− 0.181/− 0.012)	− 2.271	0.025	0.042
Cu–Zn SOD	− 0.029 (− 0.066/0.008)	− 1.556	0.123	0.015
TBARS	− 0.006 (− 0.053/0.040)	− 0.272	0.786	− 0.010

**Table 2:** A relationship between averageness (distance from average) and measures of oxidative stress tested by regression analyses.

	b (CI)	t	p	R^2^
**AVRG**				
8-OHdG	− 0.140 (− 0.253/− 0.027)	2.450	0.016	0.050
Cu–Zn SOD	− 0.054 (− 0.103/− 0.005)	2.180	0.032	0.038
TBARS	0.002 (− 0.061/0.065)	0.072	0.943	− 0.010
**SAVRG**				
8-OHdG	− 0.131 (− 0.264/0.002)	1.951	0.054	0.028
Cu–Zn SOD	− 0.056 (− 0.113/0.002)	1.923	0.057	0.027
TBARS	0.005 (− 0.068/0.079)	0.142	0.888	− 0.010

**Figure 2:**
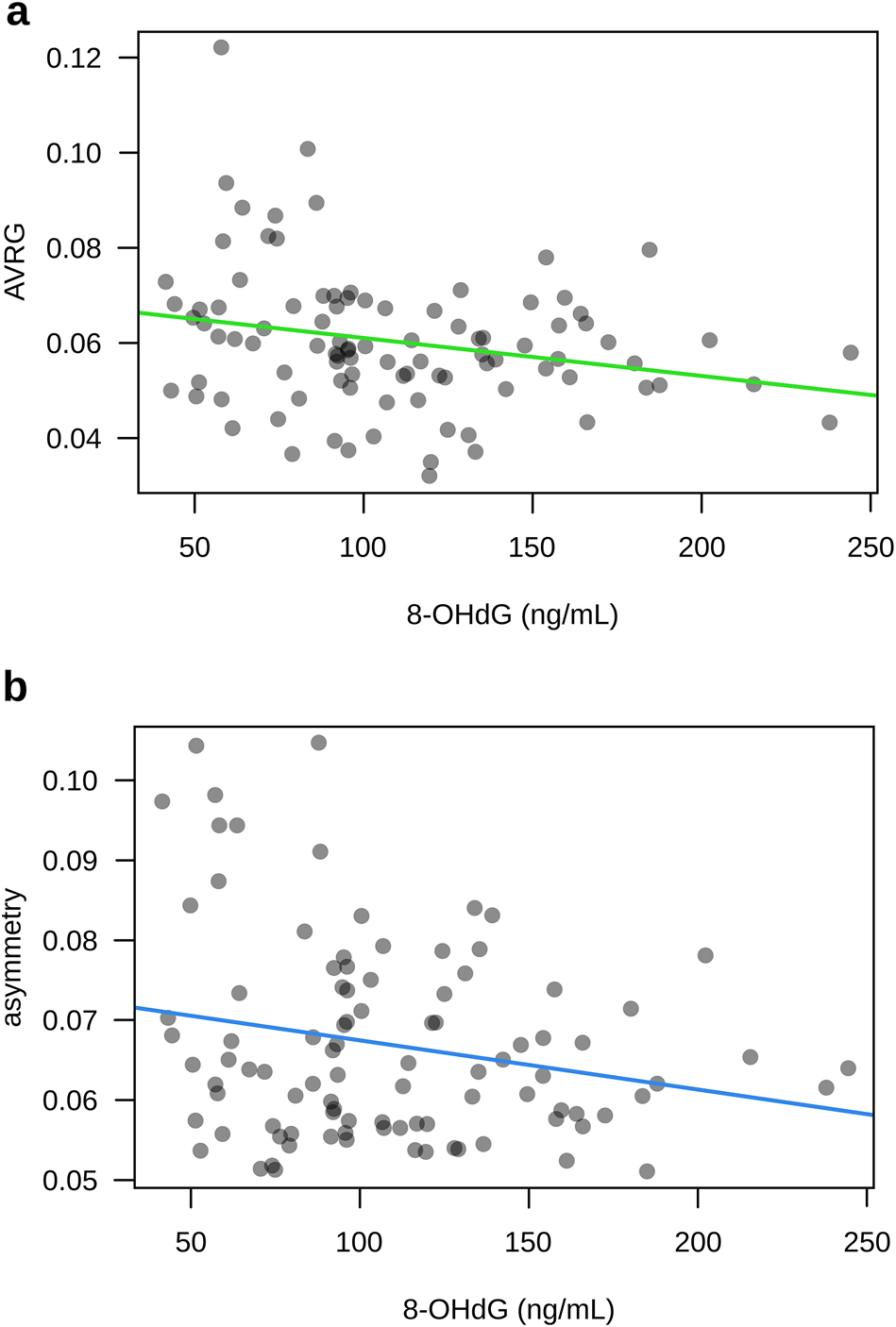
Scatter plots of the relation between 8-OHdG and (**a**) averageness (AVRG) and (**b**) facial asymmetry with a plotted regression line.

Facial symmetry showed a negative, statistically significant association with 8-OHdG, i.e., the individuals with higher levels of this OS marker tended to have less symmetrical faces (Fig. 2a). Two remaining OS markers (Cu–Zn SOD and TBARS) had no statistically significant relationship with variation in facial symmetry.

Both measures of averageness tended to correlate with 8-OHdG and Cu–Zn SOD negatively. Still, only AVRG showed a statistically significant relationship: the higher the OS marker value, the lower the measured averageness (Fig. 2b). TBARS had no statistically significant association with eithers measures of averageness.

## Discussion

Our study is the first to demonstrate significant associations between the level of oxidative stress in women and their perceived attractiveness, health, youthfulness, and symmetry. In particular, we found that female composite faces representing features of higher oxidative stress were perceived as more attractive, healthier, and younger. In contrast, faces featuring higher oxidative stress were perceived as having lower symmetry. Interestingly, the computational approach (Geometric Morphometric Modeling) did not yield any statistically significant associations. Results of the facial judgments, however, represent the first evidence for the potential cost of maintaining facial attractiveness during the lifetime and contribute to research on the relationship between perceived facial features and somatic state of the individual.

### How objective and subjective measures differ?

In our study, the specific facial morphology of women with low vs. high OS could not be identified based on Geometric Morphometric (GM) analysis. GM modeling is based on a mathematical representation of face shape and is more of an objective face space measure, than the more subjective ratings of random judges. Participants in our studies had to repeatedly detect a specific set of features that defined faces with the higher OS, whereas the sole computation of coordinates of landmarks failed to find it. Human perception may be sensitive to minuscule variation in facial features that shape analysis was not able to detect. Alternatively, perception might utilize facial traits independent of facial shape such as skin texture and tone. Thus, the variation in facial representation caused by the varying levels of OS may have been conveyed in features other than just shape.

### Possible role of oxidative shielding

Recent studies in the area of trade-offs between reproduction and maintenance resulted in the development of the *oxidative shielding* hypothesis^45^.⁠ This hypothesis posits that during sensitive periods of reproduction, females’ bodies will decrease the level of oxidative damage in selected tissues to protect themselves, gametes, and developing embryos from the negative consequences of oxidative stress. It is also proposed that the timing of this “oxidative shielding” will differ amongst taxa depending on their reproductive pattern. This suggests that increases in the level of oxidative damage could be observable between reproductive and non-reproductive periods. Although the hypothesis does not propose any specific physiological mechanism behind the shielding, evidence for oxidative shielding manifests in several animal species^46–48^⁠.

In contrast to many seasonally breeding species, human females during their reproductive years usually do not experience total reproductive suppression. In traditional populations, menstrual cycles are followed by a pregnancy, then a lactation, and then again cycles, and the next pregnancy. Therefore, an increase in oxidative damage should manifest itself mainly during the postmenopausal period. Indeed, a study of oxidative stress in response to reproductive effort conducted in humans demonstrated increased biomarkers of oxidative stress in relation to higher gravidity and parity in Polish postmenopausal women^22^. If the oxidative shielding hypothesis holds in women and oxidative damage is kept low during the reproductive period, it would explain the lack of the association between facial attractiveness and oxidative stress observed in the only one up-to-date study on young women^29^.

This hypothesis could also explain our unexpected results demonstrating higher perceived attractiveness associated with higher oxidative stress. Since maintaining attractive features is costly in terms of oxidative damage, especially in an environment scarce in resources (as it was demonstrated in several animal studies, e.g.^15, 49^), and oxidative damage must be kept low throughout the reproductive period, the cost of maintaining attractive features in women might be revealed only after menopause. Thus, “costs of beauty” remain hidden during the significant part of a woman’s life, and higher attractiveness during reproductive life related to greater oxidative damage is revealed only during the postmenopausal period.

### Additional possible proximate explanation

An additional, hypothetical proximate mechanism that may provide a possible explanation at the molecular level stems from the interpretation of the functioning of OS markers. 8-OHdG is interpreted as a marker of the efficiency with which DNA is repaired. It is possible that women who were perceived as younger and healthier in our sample were also more efficient in repairing DNA damaged by OS. This is consistent with our finding of the increased level of Cu–Zn SOD in the morphed faces of women with high OS and, in general, by the positive association between biomarkers of oxidative damage and repair. Together these results may suggest that women with more attractive faces and possibly higher biological quality efficiently handled oxidative damage to DNA by producing higher levels of SOD. However, they could not escape oxidative stress associated with the higher physiological cost of facial beauty after the reproductive period when the shielding mechanism is no longer at work. With the existing evidence, we are, however, unable to confirm this hypothesis.

### Negative relation between oxidative stress and symmetry and averageness

Facial symmetry as an indicator of general fluctuating asymmetry was hypothesized to depict developmental instability and thus serve as a proxy of lower biological quality^50^. This facial feature was also identified as a crucial component of facial attractiveness. However, more recent studies question the importance of symmetry and averageness with regards to attractiveness. For instance, Jones and Jaeger^44^ reported on the association between symmetry, averageness, and attractiveness, concluding that “the parameters that influence facial attractiveness are mostly unsupported by an overarching theory and have only a small impact on absolute attractiveness perceptions”. Similar conclusions can be drawn from Foo et al.^29^ examining the effect of several facial features, including symmetry and averageness, on perception of attractiveness and health. In this study, neither symmetry nor averageness predicted perceived attractiveness in females. Together, these results do not exclude the possibility that facial symmetry and averageness may be indicative of health or physiological state. They suggest, however, that perception of health and attractiveness based on these cues might not be as unequivocally universal as it was hypothesized previously. This reasoning provides support for the opposite directions of relationships between OS and attractiveness and OS and symmetry observed in this study.

Additionally, in study number 1, faces with higher OS were perceived as less symmetrical, which was further supported by results of computational study number 3, where measured symmetry was found to be significantly, albeit weakly, negatively related to the OS. As there was a relation between measured symmetry and OS level, the composites used to create visual stimuli in the first study also must have differed in symmetry in a manner detectable by raters. Interestingly, however, the perception of decreased symmetry did not influence the perception of increased attractiveness of high OS faces, further supporting previous results of Foo et al.^29^ and Jones and Jaeger^44^.

### Limitation and future directions

Due to the fact that data were collected in rural areas, mainly with mobile equipment, it was difficult to take photographs in lightening conditions standardized enough to allow for color and texture analysis. In light of hypotheses associating carotenoids' effect with attractiveness and oxidative damage^26^, it is possible that OS levels affect texture and pigmentation more than they do the shape of the face. However, this mechanism would not explain why High OS facial composites, created as an average of faces with highest measured OS levels were found to be more attractive, judged younger, and healthier, than Low OS composites, and not simply indifferentiable.

Another idea for a future study is to create facial composites that vary in levels of different OS markers. In this sample, the OS markers were too strongly correlated with each other to perform such an analysis. By testing varying perception and measurements of composites with three varying OS markers, one could attempt to disentangle which particular mechanism is responsible for the unexpected results of the current study 1. Additionally, in the online study number of male and female participants was not equal (i.e. less than 10% of raters were men). As men’s and women’s perception of facial attractiveness might differ, future collection of data should focus on gathering equal numbers of raters of both genders.

Finally, our results could provide a mechanistic explanation for a previously found correlation between certain types of endometriosis (a pathology putatively implicated by heightened oxidative stress levels^51^) and perceived attractiveness^52^. However, in that study the attractiveness judgement was based on “direct evaluation” of participants’ whole bodies by four (2 male and 2 female) physicians, hence correlation between facial attractiveness, either judged or computed, and such direct body evaluation is unknown. Thus, to support the association, future studies should include a greater number of judges and improved methods to evaluate facial attractiveness.

### Conclusions

This study demonstrates that faces of postmenopausal women with higher oxidative stress are rated as more attractive, healthier, and younger than faces of women with lower oxidative stress. It also shows that the only parameters that differentiate between low vs. high OS faces were symmetry and averageness, with an effect of faces being less average and less symmetrical with increasing oxidative stress (although this effect was not very strong). Together, the results of our study may provide evidence that maintaining attractiveness during lifetime incurs significant physiological costs. These “costs of beauty” remain hidden during the substantial part of a woman’s life (due to oxidative shielding during the reproductive period) and reveal themselves only after menopause in the form of increased oxidative stress.

## Supplementary information

Supplementary Information 1.

Supplementary Information 2.
