# Supplementary material for: Oxidative stress as a hidden cost of attractiveness in postmenopausal women
Source: Sci Rep. 2020 Dec 15;10:21970. doi: 10.1038/s41598-020-76627-9 (PMC7738509; doi:10.1038/s41598-020-76627-9)
Supplement: Supplementary file 2 — Supplementary Information 2. [file 41598_2020_76627_MOESM2_ESM.docx]

Electronic Supplementary Material 2

**Oxidative stress as a hidden cost of attractiveness in postmenopausal women**

Urszula M. Marcinkowska*^1^, Anna Ziomkiewicz*^2^, Karel Kleisner^3^, Andrzej Galbarczyk^1,@^, Magdalena Klimek^1^, Amelia Sancilio^4,5^, Grazyna Jasienska^1^, Richard G. Bribiescas^4^

*joint first authorship

@corresponding author

1 Department of Environmental Health, Faculty of Health Sciences, Jagiellonian University Medical College, Krakow, Poland

2 Department of Anthropology, Faculty of Biology, Jagiellonian University, Krakow, Poland

3 Department of Philosophy and History of Science, Faculty of Science, Charles University, Prague, Czech Republic

4 Department of Anthropology, Yale University, New Haven, CT, United States of America

5 Department of Anthropology, Northwestern University, Evanston, IL, United States of America

Table S1.

U Mann-Whitney test for difference in average age of women included in low and high Oxidative Stress composite.

| U | Z | p | Z adj | p | N 1 | N 2 |
| --- | --- | --- | --- | --- | --- | --- |
| 45.00000 | 0.340168 | 0.733730 | 0.340168 | 0.733730 | 10 | 10 |

Table S2.

U Mann-Whitney test for difference in Oxidative stress markers of women included in low and high Oxidative Stress composite.

| Marker | U | Z | p | Z adj | p | N 1 | N 2 |
| --- | --- | --- | --- | --- | --- | --- | --- |
| 8-OHdG | 0.00 | -3.74185 | 0.000183 | -3.74185 | 0.000183 | 10 | 10 |
| TBARS | 0.00 | -3.74185 | 0.000183 | -3.74326 | 0.000182 | 10 | 10 |
| ZnCuSOD | 0.00 | -3.74185 | 0.000183 | -3.74185 | 0.000183 | 10 | 10 |

Table S3.

Differences in the assessments of LOS faces between female and male judges

|  | Sex | N | Median | Mann-Whitney U | p |
| --- | --- | --- | --- | --- | --- |
| Health LOS | Female | 870 | 0.3 | 33222 | < 0.001 |
|  | Male | 96 | 0.4 |  |  |
| Attractiveness LOS | Female | 870 | 0.3 | 36814 | 0.054 |
|  | Male | 96 | 0.3 |  |  |
| Youthfulness LOS | Female | 870 | 0.3 | 35876 | 0.022 |
|  | Male | 96 | 0.4 |  |  |
| Symmetry LOS | Female | 870 | 0.5 | 34564 | 0.005 |
|  | Male | 96 | 0.7 |  |  |
